# Reactive Oxygen Species Rewires Metabolic Activity in Acute Myeloid Leukemia

**DOI:** 10.3389/fonc.2021.632623

**Published:** 2021-03-11

**Authors:** Andrew J. Robinson, Sara Davies, Richard L. Darley, Alex Tonks

**Affiliations:** Department of Haematology, Division of Cancer & Genetics, School of Medicine, Cardiff University, Cardiff, United Kingdom

**Keywords:** reactive oxygen species, NADPH Oxidase (NOX), acute myeloid leukemia, metabolism, redox signaling

## Abstract

Acute myeloid leukemia (AML) is a heterogeneous disease with poor clinical outcomes. We have previously shown that constitutive activation of NADPH oxidase 2 (NOX2), resulting in over-production of reactive oxygen species (ROS), occurs in over 60% of AML patients. We have also shown that increased ROS production promotes increased glucose uptake and proliferation in AML cells, mediated by changes in carbohydrate metabolism. Given that carbohydrate, lipid, and protein metabolisms are all intricately interconnected, we aimed to examine the effect of cellular ROS levels on these pathways and establish further evidence that ROS rewires metabolism in AML. We carried out metabolomic profiling of AML cell lines in which NOX2-derived ROS production was inhibited and conversely in cells treated with exogenous H_2_O_2_. We report significant ROS-specific metabolic alterations in sphingolipid metabolism, fatty acid oxidation, purine metabolism, amino acid homeostasis and glycolysis. These data provide further evidence of ROS directed metabolic changes in AML and the potential for metabolic targeting as novel therapeutic arm to combat this disease.

## Introduction

Reactive oxygen species (ROS) is the collective term for oxygen containing free radicals and other reactive molecules, including hydrogen peroxide (H_2_O_2_), which exert important cellular functions both in innate immunity and as cell signaling molecules (1). Physiologically, production of cellular ROS mainly occurs as a result of oxidative phosphorylation in the mitochondria, or *via* the transmembrane proteins, nicotinamide adenine dinucleotide phosphate (NADPH) oxidase family of enzymes (NOX) (2). In particular, NOX2, which is expressed on the plasma membrane of hematopoietic cells, generates ROS *via* the univalent reduction of molecular oxygen, producing extracellular superoxide, which rapidly dismutates to H_2_O_2_, either spontaneously or *via* the catalytic action of the enzyme superoxide dismutase (3). H_2_O_2_ is a relatively long lived ROS molecule which is able to traverse cell membranes, and this, alongside its ability to reversibly oxidize cysteine residues in the active sites of regulatory proteins, underlies its function as a cell signaling molecule (4). H_2_O_2_ plays an integral role in hematopoiesis both through direct and indirect regulation of gene expression (5, 6).

Previously, using hematopoietic stem progenitor cells as a model for hematopoiesis, we demonstrated that mutant N-RAS^G12D^ promotes ROS production *via* NADPH oxidase 2 (NOX2) (7). We further showed that over-production of NOX-derived ROS in acute myeloid leukemia (AML) promotes proliferation which is associated with defective oxidative stress signaling (8). Indeed, over 60% of AML patients show elevated levels of extracellular superoxide and H_2_O_2_, and furthermore these levels correlate with the levels of NOX2 expression (8). To understand the underlying mechanism through which ROS promotes proliferation, we previously used transcriptome profiling to identify changes in gene expression impacted by ROS over-production (6). We demonstrated that ROS mediated proliferation was attributed to changes in carbohydrate metabolism, with a key glycolytic regulator, 6-phosphofructo-2-kinase/fructose-2,6-bisphosphatase 3 (PFKFB3), acting as an important mediator of ROS. Elevated levels of PFKFB3 have been detected in numerous cancers including, colon, prostate, breast, lung, pancreatic, ovarian, kidney, and thyroid (9).

In addition to carbohydrate metabolism, there are two other main classes of molecules involved in metabolism, proteins, and lipids. These can be either catabolized to produce energy or synthesized to molecules such as nucleotides and structural proteins for the generation of cell membranes. Given that carbohydrate, lipid, and protein metabolisms are all intricately interconnected, we aimed to examine the effect of ROS on these pathways and establish further evidence that ROS rewires metabolism in AML. Using metabolomic profiling of AML cell lines (in which ROS production was inhibited by knocking down NOX2 expression) or using a cell line incubated with glucose oxidase (GOX; an enzyme that produces H_2_O_2_), we report significant metabolic alterations in sphingolipid metabolism, fatty acid oxidation (FAO), purine metabolism, amino acid homeostasis and glycolysis.

## Materials and Methods

### Materials

Key reagents and resources are provided in below.

**Table:** 

Reagent or Resource	Source	Resource Identifier (RRID) or Cat #
**Antibodies**		
NOX2-PE	MBL Life Science Nagoya, Japan,	RRID : AB_591389
IgG1-PE	MBL Life Science Nagoya, Japan	RRID : AB_1279372
		
**Chemical, Peptides, Recombinant proteins**		
Diogenes™	GeneFlow, Staffordshire U.K.	Cat # A2-0092
Diphenyleneiodonium (DPI)	Sigma-Aldrich, Poole, U.K	Cat # D2926
Glucose Oxidase	Sigma-Aldrich, Poole, U.K	Cat # G6766
Lipofectamine 3000	Invitrogen, Paisley, U.K	Cat # 11668019
7-AAD	Sigma-Aldrich, Poole, U.K	Cat # SML1633-1ML
NOX2 shRNA	Vector Builder	
		
**Experimental Models: Cell lines**		
Human: 293T	ATCC, Middlesex, U.K.	RRID : CVCL_0063
Human: Mv4;11	ATCC, Middlesex, U.K.	RRID : CVCL_0064
Human: NOMO-1	DSMZ, Germany	RRID : CVCL_1609
Human: THP-1	EACC, Salisbury, U.K.	RRID : CVCL_0006
		
**Analytical platform, Software**		
Metabolic assays	Metabolon, USA	
FCS express v6	DeNovos Software, California, U.S.A	RRID : SCR_016431

### Methods

#### Cell Culture

Cell lines were cultured according to recommended conditions at 37°C, 5% CO_2_ for all experiments. The genetic identity of the cell lines was confirmed by short tandem repeat (STR) at purchase. THP-1 and NOMO-1 cells were lentivirally transduced with shRNA complementary to *NOX2* mRNA and encoding puromycin resistance (THP-1 NOX2-KD or NOMO-1 NOX KD) and control cells (shTHP-1 or shNOMO-1) which had been transfected with shRNA coding for a non-mammalian target sequence as previously described (6). Additionally, control cells (shTHP-1/NOMO-1) were treated with the NOX inhibitor diphenyleneiodonium (100 nM) for 24 h prior to metabolomic analysis. DPI was reconstituted in DMSO and the final concentration was <0.01% DMSO. This dose has previously been shown to inhibit NOX activity without compromising cell viability (6). To mimic the effect of NOX2 generated ROS, Mv4;11 cells were treated with glucose oxidase (GOX; 10 and 20 mU/ml), which catalyzes the production of H_2_O_2_ in cell culture, for 24 h prior to metabolomic analysis. Control cells were treated with 0.002% v/v DMSO (Vehicle control). Viability was tested using 7-AAD (1 μg/ml) and analyzed using flow cytometry; viable cells were used in subsequent superoxide and metabolic assays.

#### Determination of NOX2 Expression

To determine expression levels of NOX2, cells were incubated with NOX2 PE conjugated antibody (5 ng/μl) or an isotype matched control (MBL), incubated for 45 min at 4°C and analyzed by flow cytometry. All flow cytometric data were acquired using an Accuri C6 flow cytometer (Becton Dickinson, U.K.). A minimum of 3,000 events was collected in the region of interest. Data analysis was performed using FCS express v6.

#### Detection of Superoxide

Cell cultures were adjusted for viable cell number and superoxide measurement carried out using the chemiluminescent probe Diogenes as previously described (7).

#### Metabolomics

Metabolomic analysis was performed on quadruplicate samples of the AML cell lines THP-1, NOMO-1 and Mv4;11 by Metabolon™ (http://www.metabolon.com). Cell line samples were analyzed using ultra-high-performance liquid chromatography mass spectrometry (UPLC-MS), utilizing Waters ACQUITY UPLC and Thermoscientific Q-Exactive high resolution mass spectrometer interfaced with a heated electrospray ionization source and Orbitrap mass analyzer. Raw data was extracted, peaks identified, and quality control processed using proprietary Metabolon™ hardware, software, and biochemical library database. Following normalization to Bradford protein concentration, log transformation and imputation of missing values with the minimum observed value for each compound, Welch unequal variance two-sample t test was performed to identify significant differences between the experimental groups. To account for potentially high false discovery rate (because of multiple comparisons), a *q*-value was also calculated, where a lower *q*-value is an indication of higher confidence in the result.

## Results

### ROS Induce Global Changes in Metabolism in AML Cell Lines

**Figure 1:**
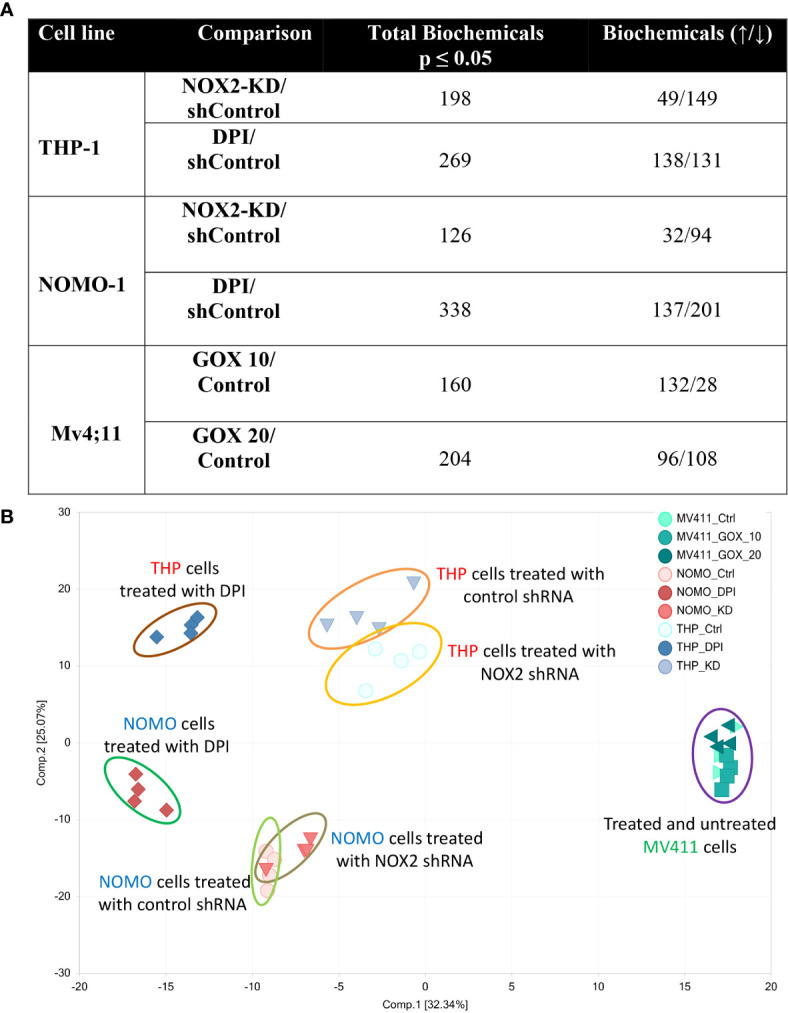
Production of ROS is associated with global changes in metabolism in AML cell lines. Data from global biochemical profiling of shTHP-1, shNOMO-1 and Mv4;11 AML cell lines. THP-1/NOMO-1 cells with NOX2 knocked down using shRNA (NOX2-KD), compared to control cells infected with non-mammalian targeting control shRNA (control) or 100 nM DPI, a NOX inhibitor. Mv4;11 cells were treated with 10 mU/ml or 20 mU/ml GOX (compared to untreated control). **(A)** Summary of the numbers of biochemicals that achieved statistical significance (*p ≤ 0.05) analyzed by Welch’s two sample t-test. **(B)** Principal components analysis (PCA) of global biochemical profiling of AML cell lines. Sh control cells were treated with 100 nM DPI for 24 h or NOX2 was KD using shRNA (n = 4). Mv4;11 cells were treated with 10 or 20 mU/ml of GOX for 24 h (n = 4).

Our previous study shows that NOX2 derived ROS in AML patient blasts increases glucose uptake (6). These changes in carbohydrate metabolism could also be induced by the addition of GOX (providing a source of exogenous H_2_O_2_) in an AML cell line (Mv4;11) that does not generate NOX2 derived ROS. To establish further evidence that ROS affects metabolism in AML, we have now analyzed the whole metabolome of AML cell lines producing different levels of NOX-2 derived ROS. Since Mv4;11 cells have very low levels of ROS, we treated these cells with GOX. Conversely, we have also analyzed the metabolome of lines generating NOX2 derived ROS (THP-1 and NOMO-1) and have examined the impact of knockdown or inhibition of NOX2 in this context using shRNA vectors and DPI, respectively. To compare NOX2-KD to an appropriate control, we created control lines infected with non-mammalian target (labeled ‘sh’). The levels of DMSO in treated samples were less than 0.01%. Given the very low levels of DMSO, we did not treat control samples with this proportion of DMSO as the effects would be negligible. The knockdown of NOX2 expression/superoxide production (>90% reduction) in THP-1 NOX2 KD as well as the impact of DPI on THP-1 cells has been previously described (6). **Supplemental Figure S1** shows knockdown of NOX2 and reduced superoxide production in NOMO-1 cells. Analysis of the impact of NOX2 knockdown or inhibition on the global biochemical metabolic profile whose levels were significantly altered in THP-1 and NOMO-1 cells is shown in **Figure 1**. Treatment of Mv4;11 cells with H_2_O_2_ (mediated by incubation with GOX) resulted in several significant changes of biochemical metabolites when cultured with 10 and 20 mU/ml, respectively. Analysis of the overall biochemical variations between each sample was performed using principal component analysis (PCA) (**Figure 1B**). This analysis revealed significant separations based on the individuality of cell lines and treatment conditions (*i.e.*, the samples from each AML cell line clustered relatively close to each other). Due to the limited use of three cell lines, it was not possible to correlate changes with cell line abnormalities (*e.g*. mutational or genotypic analysis). However, NOX2 KD and those cells treated with DPI showed significant differences of biochemical metabolite levels compared to shTHP-1/NOMO-1 controls. The largest effects were observed with DPI inhibition. The global differences between Mv4;11 cells treated/untreated with GOX were much more modest (**Figure 1B**). This data is supported by hierarchical clustering where samples also clustered according to genotype and treatment status (**Supplemental Figure S2**).

Furthermore, Random Forest (RF) analyses of cellular metabolic profiles (**Supplemental Figure S3A, B**) showed 100% prediction accuracy when differentiating shTHP-1 or shNOMO-1 control cells both from those treated with DPI and cells with NOX2-KD cells; as compared to 33.3% by random chance alone. Prediction accuracy in differentiating Mv4;11 untreated cells from those treated with GOX was 91.7% (**Supplemental Figure S3C**). The high predictive accuracies of the analyses are consistent with the large number of statistically significant differences between the groups (**Figure 1A**). Taken together, these data indicate that while cell line origin was the largest determining factor in changes in global biochemical metabolite variation, culture in a ROS environment affects the metabolome or global biochemical metabolite composition.

### ROS Alters Metabolism Linked to Fatty Acid Oxidation in AML Cell Lines

Across all conditions tested above, RF analysis showed consistent changes which were observed in lipid metabolism (**Supplemental Figure S3**). To determine the NOX2 mediated ROS effects on lipid metabolism we have compared the common and unique metabolic changes in THP-1 and NOMO-1 cells where NOX2 levels were knocked down (or inhibited) and Mv4;11 cells treated with GOX. These cell lines showed significant changes in sphingolipid metabolism and FAO (**Table 1**). Knock-down of NOX2 in THP-1 and NOMO-1 cells significantly decreased levels of sphingomyelins (phospholipids composed of ceramide and phosphocholine) as well as sphingosine and, in THP-1, sphinganine metabolites which are involved in their synthesis and degradation (10). Levels of sphingosine and sphinganine were also significantly decreased in these cells treated with DPI (**Table 1**). Conversely, Mv4;11 cells, significant increases in sphingomyelin levels were observed upon treatment with GOX (**Table 1**). Together these data suggest that ROS levels are in important in regulating sphingolipid synthesis and/or degradation.

**Table 1:** Exposure of AML cell lines to ROS is associated with global changes in sphingolipid metabolism.

Biochemical name	THP-1		NOMO-1		Mv4;11	
	NOX2 KD *vs* shControl	DPI *vs* shControl	NOX2 KD *vs* Control	DPI *vs* shControl	GOX 10 mU/ml *vs* Control	GOX 20 mU/ml *vs* Control
N-palmitoyl-sphinganine (d18:0/16:0)	**0.68**	**0.49**	**1.30**	**0.81**	**2.27**	**2.88**
sphinganine	**0.82**	**0.47**	1.10	**0.30**	1.06	0.99
phytosphingosine	**0.65**	0.80	**0.81**	**0.63**	1.12	1.13
palmitoyl sphingomyelin (d18:1/16:0)	**0.80**	**1.22**	**0.85**	0.98	1.09	0.89
stearoyl sphingomyelin (d18:1/18:0)	**0.82**	1.10	**0.65**	0.96	**1.31**	1.04
sphingomyelin (d18:1/18:1, d18:2/18:0)	0.89	1.16	**0.61**	0.96	**1.39**	1.14
sphingosine	**0.62**	**0.37**	**0.70**	**0.30**	1.13	1.00
N-palmitoyl-sphingosine (d18:1/16:0)	**0.76**	**1.68**	**0.88**	**1.27**	1.08	0.88
sphingomyelin (d18:1/14:0, d16:1/16:0)*	**0.80**	**1.32**	0.88	**1.22**	1.15	0.96
sphingomyelin (d18:2/14:0, d18:1/14:1)*	**0.77**	**1.24**	0.85	1.16	**1.45**	**1.37**
sphingomyelin (d18:1/24:1, d18:2/24:0)*	**0.81**	1.17	0.85	0.85	**1.32**	1.06
sphingomyelin (d18:2/16:0, d18:1/16:1)*	**0.78**	1.07	0.88	0.91	**1.35**	1.19
sphingomyelin (d18:1/20:1, d18:2/20:0)*	0.81	0.92	**0.76**	**1.21**	**1.76**	**1.43**
behenoyl sphingomyelin (d18:1/22:0)*	0.83	1.11	0.83	1.08	**1.44**	1.14
sphingomyelin (d18:1/22:1, d18:2/22:0, d16:1/24:1)*	**0.79**	1.09	0.89	**1.26**	**1.72**	**1.43**
sphingomyelin (d18:1/20:0, d16:1/22:0)*	**0.82**	1.00	0.86	1.16	**1.46**	**1.20**
palmitoyl dihydrosphingomyelin (d18:0/16:0)*	1.09	0.93	1.18	**0.64**	**2.23**	**2.10**
sphingomyelin (d18:1/15:0, d16:1/17:0)*	**0.77**	1.14	0.87	1.07	**1.19**	1.04
sphingomyelin (d18:1/21:0, d17:1/22:0, d16:1/23:0)*	**0.79**	0.96	**0.79**	**1.44**	1.70	**1.52**
sphingomyelin (d18:2/23:0, d18:1/23:1, d17:1/24:1)*	**0.77**	1.09	**0.74**	1.09	**1.59**	**1.35**
sphingomyelin (d18:2/24:1, d18:1/24:2)*	**0.74**	1.14	**0.78**	0.97	**1.60**	**1.33**
tricosanoyl sphingomyelin (d18:1/23:0)*	**0.80**	1.16	**0.76**	1.11	**1.70**	**1.47**
sphingomyelin (d18:1/17:0, d17:1/18:0, d19:1/16:0)	0.74	1.03	**0.71**	1.09	**1.39**	**1.23**
glycosyl-N-stearoyl-sphingosine	**0.70**	0.97	**0.70**	0.97	0.94	**0.70**
glycosyl-N-palmitoyl-sphingosine	**0.62**	0.91	0.90	0.99	1.10	0.91
lactosyl-N-palmitoyl-sphingosine	0.86	0.97	0.93	0.90	1.00	**0.83**

Table shows mean fold changes in fatty acid oxidation in THP-1 and NOMO-1 cells with NOX2 KD when compared to controls (transduced with control shRNA vector). Changes in sphingolipids were also compared in shTHP-1 and shNOMO-1 cells treated with DPI (100 nM) for 24 h or Mv4;11 cells treated with increasing GOX units/ml. Data shows relative values following normalization to protein concentration, log transformation and imputation of missing values with the minimum observed value for each compound (n = 4). Color boxes indicate ratios and p-values for each comparison: boxes shaded dark green indicate significant decreases and boxes shaded dark red indicate significant increases; statistical significance was performed using Welch’s two sample t-test (P < 0.05). Boxes shaded light green or red indicate decreases or increases, respectively and approaching significance (p < 0.1). * Indicates compounds that have not been officially confirmed based on a standard, but we are confident in its identity.

It has previously been reported that NOX2 inhibition leads to increased FAO (11) and that FAO can be an important method of ATP production in solid tumors experiencing metabolic stress (12, 13). Consistent with this, our data showed that knock-down of NOX2 in THP-1 and NOMO-1 or treatment of cells with DPI led to significant decreases in long chain acylcarnitines; metabolites which are consumed during FAO though reciprocal changes were not seen in Mv4;11 cells treated with GOX (**Table 2**). NOX2 KD in THP-1 cells displayed significant decreases in several 3-hydroxy fatty acids (intermediates formed during *β*-oxidation) and in free carnitine and its metabolic precursor (deoxycarnitine). Many of the latter metabolites were lower in the NOMO-1 NOX2 KD cells but fell below the cut-off for statistical significance (**Table 2**). Taken together, these data suggest that ROS affects the transport and oxidation of fatty acids.

**Table 2:** Exposure of AML cell lines to ROS is associated with global changes FAO metabolism.

Biochemical name	THP-1		NOMO-1		Mv4;11	
	NOX2 KD vs Control	DPI vs shControl	NOX2 KD vs Control	DPI vs shControl	GOX 10mU/mL vs Control	GOX 20mU/mL vs Control
hexanoylcarnitine	**0.16**	**0.05**	**0.29**	**0.04**	**0.50**	**0.29**
octanoylcarnitine	**0.27**	**0.27**	**0.19**	**0.12**	2.08	0.78
laurylcarnitine	**0.56**	**0.19**	**0.48**	**0.21**	0.83	**0.69**
myristoylcarnitine	**0.48**	**0.23**	**0.53**	**0.21**	0.76	**0.65**
palmitoylcarnitine	**0.45**	**0.18**	**0.58**	**0.15**	**0.66**	**0.56**
stearoylcarnitine	**0.40**	**0.06**	**0.74**	**0.09**	**0.63**	**0.57**
linoleoylcarnitine	**0.12**	**0.38**	1.26	0.74	1.09	**0.63**
oleoylcarnitine	**0.34**	**0.40**	**0.56**	**0.24**	1.03	**0.68**
myristoleoylcarnitine	**0.45**	**0.39**	**0.46**	**0.34**	1.08	0.79
suberoyl carnitine	**0.87**	**0.68**	**1.20**	**0.88**	0.84	0.92
deoxycarnitine	**0.57**	1.09	**0.85**	**0.68**	0.86	**0.69**
carnitine	**0.62**	0.96	**0.90**	**0.80**	0.94	**0.74**

Table shows mean fold changes in fatty acid oxidation in THP-1 and NOMO-1 cells with NOX2 KD when compared to controls (transduced with control shRNA vector). Changes in sphingolipids were also compared in shTHP-1 and shNOMO-1 cells treated with DPI (100 nM) for 24 h or Mv4;11 cells treated with increasing GOX units/ml. Data shows relative values following normalization to protein concentration, log transformation and imputation of missing values with the minimum observed value for each compound (n = 4). Color boxes indicate ratios and p-values for each comparison: boxes shaded dark green indicate significant decreases and boxes shaded dark red indicate significant increases; statistical significance was performed using Welch’s two sample t-test (P < 0.05). Boxes shaded light green or red indicate decreases or increases, respectively and approaching significance (p < 0.1).

### ROS Alters Purine and Amino Acid Homeostasis in NOX2 KD and DPI Treated AML Cell Lines

**Figure 2:**
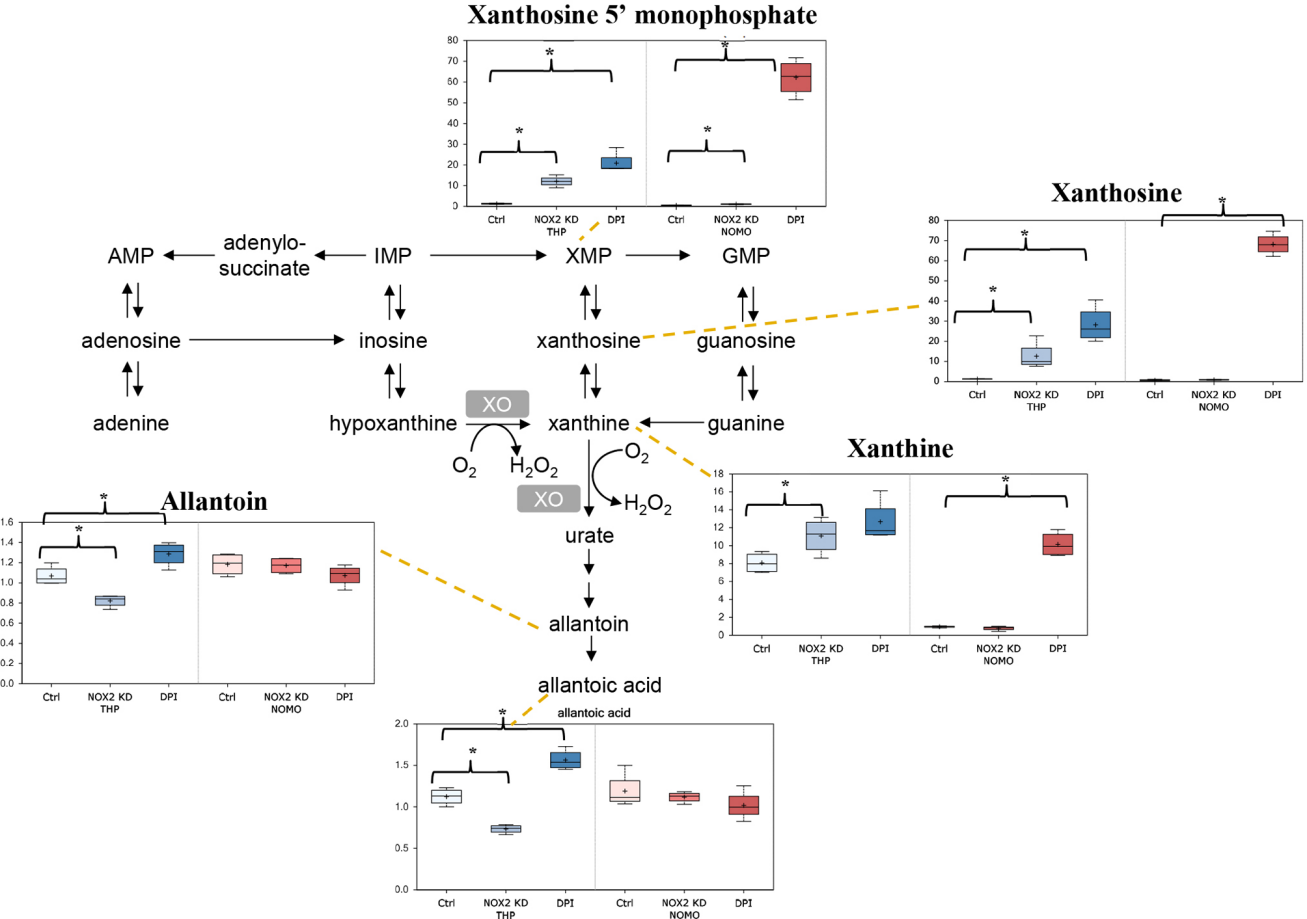
Alterations in purine metabolism in NOX2 KD and DPI-Treated AML cell lines. Data from global biochemical profiling of shTHP-1 and shNOMO-1 cells with NOX2 KD or treated with DPI (100 nM) for 24 h followed by analysis by Metabolon™. Levels of biochemicals normalized to total protein in purine metabolism. Statistical significance analyzed by Welch’s two sample t-Test (n = 4 per group) and significance denoted by *, P < 0.05. Ctrl, untreated control cells; XMP, xanthosine 5′-monophosphate; GMP, guanosin-monophosphate; XO, xanthine oxidase. THP-1 cells are color coded (blue) and NOMO-1 cells color coded Red. Y axis is scaled intensity.

It is well established that ROS contributes to enhanced proliferation of leukemia cells including the cell lines assayed in this study (6–8). Analysis of our data showed that reduction of ROS levels in THP-1 cells either through NOX2 KD or DPI treatment, resulted in several alterations in nucleotide metabolism. As shown in **Figure 2**, notable changes in purine catabolic/salvage pathway were observed. THP-1 NOX2 KD cells exhibited significant increases in xanthine, xanthine 5′-monophosphate (XMP), and xanthosine, while NOMO-1 cells with reduced levels of NOX2/ROS also showed significant increases in XMP. In parallel with these changes, THP-1 NOX2 KD cells also exhibited decreases in allantoin and allantoic acid, metabolites that can be derived from urate (the end-product of purine catabolism), suggesting that increased Xanthine metabolites are being diverted to adenosine/guanosine synthesis. In addition, treatment of Mv4;11 with GOX showed a significant increase in xanthine (**Supplemental Metabolomics File**). While XMP and xanthosine levels were increased and a reduction in allantoin and allantoic acid was observed, these levels were not statistically significant (**Supplemental Metabolomics File**). Taken together, these changes are consistent with alterations in purine utilization and degradation rates.

AML blasts producing significant levels of ROS show increased levels of metabolites associated with nucleotide metabolism (6). Significant reductions in the levels of numerous amino acids were observed in THP-1 cells in which NOX2 was knocked down or where ROS production was inhibited by DPI (**Table 3**). In the shNOMO-1 cell line, similar patterns of amino acid metabolite levels were observed when the cells were treated with DPI. However, NOX2 KD did not elicit a change when compared to controls. Decreases were also observed in select dipeptides (short polymers of amino acids typically derived *via* protein degradation) (**Supplemental Metabolomics File**). Treatment of Mv4;11 cells with H_2_O_2_ showed significant increases, at the lower (though not higher) GOX dosage (**Table 3**). Together, these data are consistent with the notion where increased amino acid production are recycled into metabolic and biosynthetic pathways necessary for increased proliferation (14).

**Table 3:** Exposure of AML cell lines to ROS is associated with global changes in amino acid metabolism.

Biochemical name	THP-1		NOMO-1		MV4;11	
	NOX2 KD vs Control	DPI vs shControl	NOX2 KD vs Control	DPI vs shControl	GOX 10mU/ml vs Control	GOX 20mU/ml vs Control
Glycine	**0.75**	**1.31**	1.02	**0.84**	1.11	**0.85**
Serine	**0.78**	**0.37**	**1.19**	**0.63**	1.09	**0.72**
Threonine	**0.77**	0.83	**1.10**	**0.51**	**1.28**	1.00
Alanine	**0.75**	**0.57**	1.19	**0.43**	0.98	**0.73**
Asparagine	**0.67**	**0.64**	**1.25**	**0.48**	1.13	0.86
Glutamate	**0.75**	0.95	0.95	0.90	1.06	**0.82**
Glutamine	**0.16**	**1.64**	**1.27**	**0.42**	1.14	**0.73**
Histidine	**0.81**	**0.74**	**1.16**	**0.72**	**1.28**	0.97
Phenylalanine	**0.79**	**0.51**	1.06	**0.65**	**1.18**	0.91
Tryptophan	**0.80**	**0.63**	1.12	**0.70**	**1.23**	1.03
Leucine	**0.77**	**0.64**	1.07	**0.71**	1.16	0.86
Methionine	**0.83**	**0.63**	1.14	0.86	**1.20**	**0.82**
Cysteine	**0.52**	1.08	**0.83**	**0.79**	**1.19**	0.97
Proline	**0.74**	0.89	**0.88**	**0.71**	**1.23**	1.03
Aspartate	**1.38**	**0.54**	**0.77**	**0.41**	**0.84**	**0.64**
Arginine	0.93	**1.32**	1.06	**0.86**	1.09	**0.78**
Isoleucine	0.82	0.89	**1.13**	**0.84**	**1.28**	1.03
Valine	0.85	**0.73**	1.06	**0.73**	**1.28**	0.95
Lysine	0.86	**0.84**	0.91	**0.63**	1.15	0.89
Tyrosine	**0.74**	**0.47**	**1.11**	**0.68**	1.18	0.92

Table shows mean fold changes in amino acids in THP-1 and NOMO-1 cells with NOX2 KD when compared to controls (transduced with control shRNA vector). Changes in sphingolipids were also compared in shTHP-1 and shNOMO-1 cells treated with DPI (100 nM) for 24 h or Mv4;11 cells treated with increasing GOX units/ml. Color boxes indicate ratios and p-values for each comparison: data shows relative values following normalization to protein concentration, log transformation and imputation of missing values with the minimum observed value for each compound (n = 4). Boxes shaded dark green indicate significant decreases and boxes shaded dark red indicate significant increases; statistical significance was performed using Welch’s two sample t-test (P < 0.05). Boxes shaded light green or red indicate decreases or increases, respectively and approaching significance (p < 0.1).

### ROS Alters the Glycolytic Metabolites Pyruvate and Lactate in AML Cell Lines

**Figure 3:**
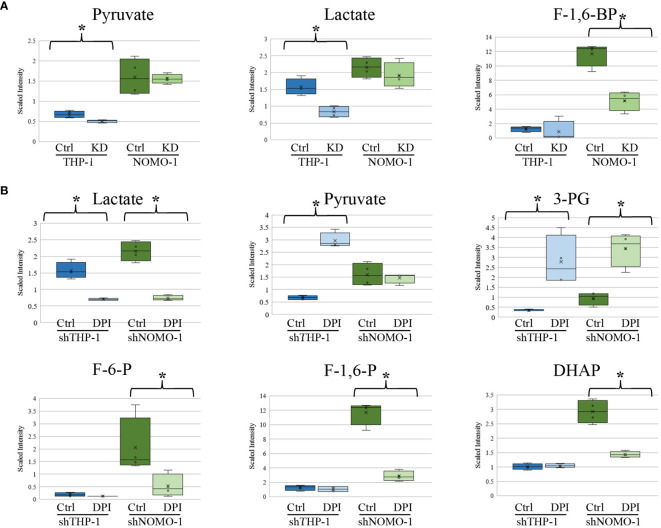
Changes in glycolytic intermediates in AML derived cell lines with NOX2 KD or treated with DPI. Significant changes in glycolytic metabolites in **(A)** shTHP-1 and shNOMO-1 cells with NOX2 KD. **(B)** shTHP-1 and shNOMO-1 cells treated with DPI (100nM) for 24h. Data shows relative values following normalization to protein concentration, log transformation and imputation of missing values with the minimum observed value for each compound (n = 4). Box plots represent median quartile ranges, x represents mean value. * denotes p < 0.05 = as analyzed by Welch’s two sample t-test. THP-1 cells are color coded (blue) and NOMO-1 cells color coded green.

We previously found that AML blasts with high levels of ROS showed significantly higher levels of glucose, glucose-6-phosphate, and fructose-6-phosphate (F-6-P) than AML blasts exhibiting low levels of ROS (6). When THP-1 and NOMO-1 cells with NOX2 KD were compared to control cells, they exhibited several alterations in metabolites linked to glucose utilization. While no significant changes in the above metabolites were observed upon modulation of ROS levels, other changes observed were consistent with a role for ROS in promoting glycolysis. The levels of pyruvate and lactate were significantly lower (1.3 and 1.9-fold respectively) in THP-1 cells with NOX2 KD. In NOMO-1 cells NOX2 KD induced a significant, 2.3-fold decrease in the glycolytic intermediate fructose-1,6-bisphosphate (F-1,6-BP) indicating decreased flux through the glycolytic pathway arising from inhibition of ROS production (**Figure 3A**). Consistent with this data, shTHP-1 cells treated with DPI also showed a significant 2.2-fold decrease in lactate levels. Surprisingly, some changes were not supportive of the role of ROS promoting glycolysis. Significant 4.4-fold increases in pyruvate, 8.1-fold increase in 3-phosphoglycerate (3-PG) were observed in THP-1 cells treated with DPI (**Figure 3B**). A significant increase in 3-PG (3.6-fold) was also observed in shNOMO-1 cells treated with DPI, while significant decreases were observed in F-6-P (3.8-fold), F-1,6-BP (four-fold), dihydroxyacetone phosphate (DHAP; two-fold) and lactate (2.9-fold) (**Figure 3B**). Taken together, these data are consistent with ROS modulated changes in biochemical levels within the glycolytic pathway.

## Discussion

Previous work by our group linked mutational RAS activation with increased NOX2 derived ROS production and cellular proliferation in normal human hematopoietic cells (7). This was supported by further studies on AML patient blasts and AML cell lines which demonstrated an association of proliferation with NOX2 derived ROS (8). Indeed, a causal link between ROS and relapse has also been established (15). Further, FLT3-ITD (and subsequent signaling), another common mutation in AML has also been shown to increase levels of ROS which were associated with increased DNA double strand breaks (16). Elevated ROS levels have also been observed in other hematological malignancies including acute lymphoblastic leukemia and chronic myeloid leukemia patient samples (17). Besides the roles in DNA damage and cell death, there is increasing evidence regarding ROS as a signaling molecule. Redox signaling can affect transcription factor activity involved in metabolic regulation, such as HIF1*α*, STAT3, and NF-*κ*B (18). More recently, we identified that ROS specifically led to changes in mRNA expression levels of several metabolic enzymes including glycolytic genes (6). We now show here using global metabolomic profiling, the impact knocking down or inhibiting NOX2 and conversely the effect of exogenous H_2_O_2_ on global cellular metabolism. To address this, we made used of two AML cell lines (THP-1 and NOMO-1) that constitutively produce extracellular superoxide (ROS) and a cell line with negligible ROS production (Mv4;11). These models permitted the reciprocal approach to reduce ROS levels (by knocking down NOX2) or to add exogenous ROS (H_2_O_2_) by incubating cells with GOX.

When NOX2 was knocked down in THP-1 and NOMO-1 cell lines, both cell types exhibited alterations in metabolites linked to FAO and complex lipid homeostasis. Treatment of both these cell lines with DPI also resulted in changes consistent with the effect of NOX2 derived ROS on metabolites linked to glucose utilization and amino acid homeostasis. These ROS induced changes in concentrations of glycolytic metabolites are commensurate with the idea that ROS induces increase in glucose uptake in these cell lines which leads to metabolic changes and redox adaptation that supports the enhanced proliferation of leukemia cells (6). However, glycolysis is only one component of cellular metabolism, with many other metabolic pathways feeding into and branching off from glycolytic intermediates, such as FAO. Support for the changes observed in lipid metabolism here can also be found in recent metabolomic studies in pancreatic ductal adenocarcinomas, which have identified sphingolipids as relevant biomarkers in this disease (19).

THP-1 NOX2-KD cells and THP-1 cells treated with DPI exhibited several unique changes, namely, alterations in metabolites linked to purine metabolism and amino acid homeostasis. Analogous changes were not observed in NOMO-1 cells with NOX2 KD. NOMO-1 cells generate significantly larger amounts of ROS than THP-1 cells (**Supplemental Figure S1**) and given that the knock-down of NOX2 in these cells was only partial, it may be that cellular ROS remained at high enough levels in these cells to prevent equivalent changes occurring. Interestingly, NOMO-1 cells treated with DPI and exhibiting lower levels of ROS than those with NOX2 knocked down showed similar changes in purine metabolites and amino acid levels to those in the THP-1 cells. Additionally, it is worth noting that changes in purine synthesis and catabolism could in themselves influence ROS production, as H_2_O_2_ is produced as a co-product by the enzyme xanthine oxidase (XO; the enzyme responsible for metabolizing xanthine to urate). In addition to these changes, THP-1 and NOMO-1 cells also exhibited decreases in the purine synthetic intermediate AICA ribonucleotide and increases in the pyrimidine synthetic intermediate orotate (**Supplemental Metabolomics File**).

The data generated from the addition of GOX to Mv4;11 cell line was more equivocal particularly at the higher dose of GOX. Addition of GOX at the lower (10 mU/ml) dose demonstrated changes to both sphingolipid metabolism and amino acid homeostasis consistent with the data generated in the THP-1 and NOMO-1 cell lines. Culture with GOX did not dose dependently increase the various sphingolipids and other sphingomyelins. It suggests higher GOX levels maybe toxic, but we did not observe changes in cell viability. Alternatively, it is interesting to speculate that higher levels of GOX are activating a negative feedback loop. Little effect was observed on purine synthesis and the impact on FAO. FAO is known to be negatively correlated with ROS production (20) and is an important source of NADPH. NADPH is also generated *via* the pentose phosphate pathway (PPP) and serine synthesis pathway, and discrepancies here may simply be reflective of differing relative cellular utilization of alternative antioxidant generating pathways. Overall, the more modest changes in this cell line may be reflective of the smaller variation between the untreated and treated samples, as revealed by the PCA analysis (**Figure 1B**), when compared with those observed in the other two cell lines.

The biochemical changes arising from the DPI treatment noted several common alterations; however, there were some degree of differences within the THP-1 and NOMO-1 cells. Specifically, both cell lines exhibited alterations in metabolites linked to glucose utilization, TCA cycle activity, lipid availability, nucleotide turnover, nicotinamide metabolism, and amino acid homeostasis. It is recognized that at micro-molar concentrations, DPI inhibits not only NOX but also mitochondrial respiration through the inhibition of NAPDH cytochrome P450 oxidoreductase, as well as, nitric oxide synthase, and xanthine oxidase [reviewed in (21)]. Additionally, it has been reported (22) that DPI inhibits not only the TCA but also the PPP, the first step of which regenerates NADPH, an important reducing agent for ROS. Inhibition of the citric acid cycle could potentially explain increases in extracellular lactate as an accumulation of pyruvate (the final product of glycolysis) would also generate proportional increases in the concentration of intracellular lactate. However, the levels of DPI used (in nanomolar range) over the time course of incubation (24 h) does not significantly affect cell viability or mitochondrial superoxide production (7). Further, it has also been suggested that at nano-molar concentrations, inhibition of NOX but not mitochondrial respiration is observed (23).

Otto Warburg initially ascribed his observation that cancer cells exhibited increased aerobic glycolysis to defective mitochondrial function in these cells (24), although a number of subsequent studies have shown functional mitochondria is important in cancer cell metabolism [reviewed in (25)]. The citric acid cycle commences from the reaction of acetyl CoA with oxaloacetate to form citrate. Acetyl CoA is generated following the decarboxylation of pyruvate catalyzed by the enzyme pyruvate dehydrogenase, while oxaloacetate is regenerated from the citric acid cycle. Importantly the metabolic step which converts succinate to fumarate involves the reduction of flavin adenine dinucleotide which generates the proton gradient necessary for oxidative phosphorylation. Furthermore, it has been shown that while hematopoietic stem cells require fumarate hydratase (the enzyme that catalyzes this step) for self-renewal and maintenance, leukemia stem cells do not (26). Therefore, ROS induced changes in fumarate levels may be indicative of changes in the cellular rates of oxidative phosphorylation. Analysis of the mass spectrometry data by Metabolon™ showed no significant changes in fumarate levels in either THP-1 or NOMO-1 cells with NOX2 KD, but significant decreases were observed in DPI treated cells (**Supplemental Figure S4**). It should be noted that decreases in fumarate metabolites could have arisen from DPI’s inhibitory effect on flavoproteins independent of NOX. Taken together, these data suggest that genetic knockdown of NOX2 in THP-1 and NOMO-1 cells does not affect the rate of oxidative phosphorylation.

In summary, exposure of cells to NOX2 derived ROS is consistent with cellular alterations in protein degradation rates, amino acid utilization, lipid metabolism and energy production. Changes of this nature may correlate with alterations in growth and proliferation rates.

## Data Availability Statement

The original contributions presented in the study are included in the article/**Supplementary Material**. Further inquiries can be directed to the corresponding author.

## Author Contributions

Conceptualization, AT and RD. Investigation, AR, SD, RD, and AT. Writing—Original Draft, AR and AT. Writing—Review & Editing, AT, RD, and AR. Funding acquisition, AT and RD. Resources, SD. Supervision, AT, RD, and SD. All authors contributed to the article and approved the submitted version.

## Funding

This work was supported by grants from Tenovus Cancer Care (AR: PhD2013/L19) and Blood Cancer UK (13029). Welcome ISSF supported AT and RD.

## Conflict of Interest

The authors declare that the research was conducted in the absence of any commercial or financial relationships that could be construed as a potential conflict of interest.
