# Supplementary material for: Reactive Oxygen Species Rewires Metabolic Activity in Acute Myeloid Leukemia
Source: Front Oncol. 2021 Mar 11;11:632623. doi: 10.3389/fonc.2021.632623 (PMC7993200; doi:10.3389/fonc.2021.632623)
Supplement: Supplementary file 1 [file DataSheet_1.pdf]

Supplementary Figures

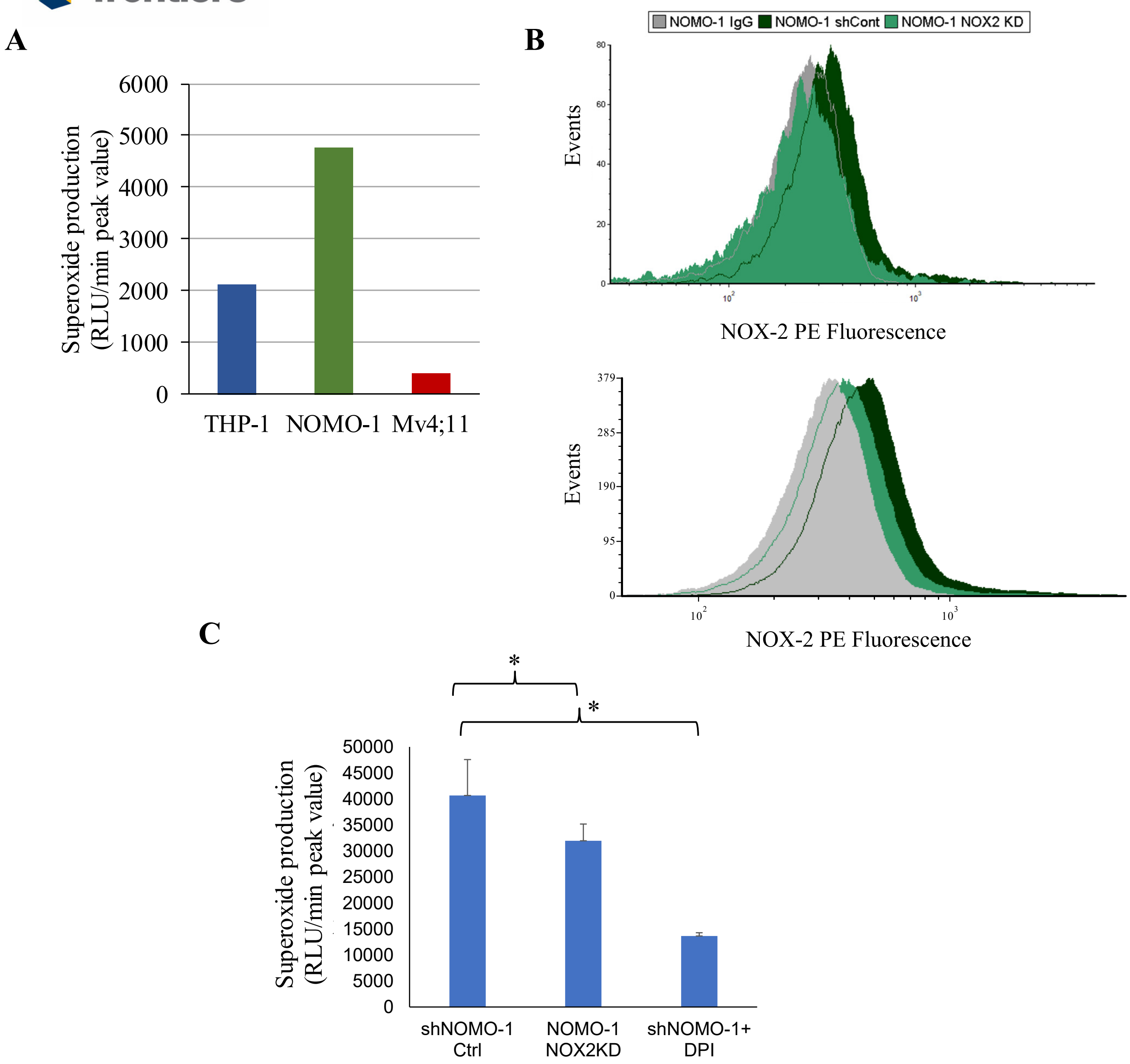

Supplemental Figure S1. (A) Representative superoxide production in AML cell lines. Peak superoxide production (relative luminescent units/min (RLU/min)) in a panel of AML derived cell lines measured using Diogenes^{TM} (n=1). **(B)** Cell surface NOX2 expression in NOMO-1 (Parental control; dark green) and NOMO-1 NOX2-knock down (KD) (light green) determined using an anti-NOX2-PE conjugated antibody and compared to IgG_1 isotype parental control (grey). X-Axis shows fluorescence intensity. Y-axis depicts number of events at given fluorescence intensities. Data is normalized to peak height based on viable (7AAD-FL3) and forward and side scatter parameters (FSC/SSC) with a minimum of 3,000 events collected in the region of interest. Top panel show NOX2 expression immediately following transduction. Bottom panel shows expression of NOX-2 immediately prior to metabolomics. **(C)** Superoxide production (RLU) in NOMO-1 cells infected with shRNA control (shNOMO-1), NOMO-1 NOX2-KD, and shNOMO-1 treated with DPI (100nM) for 24 h (light green). Data represents mean± 1SD (n=4). * P<0.05 analyzed by ANOVA and Tukeys post-hoc test.

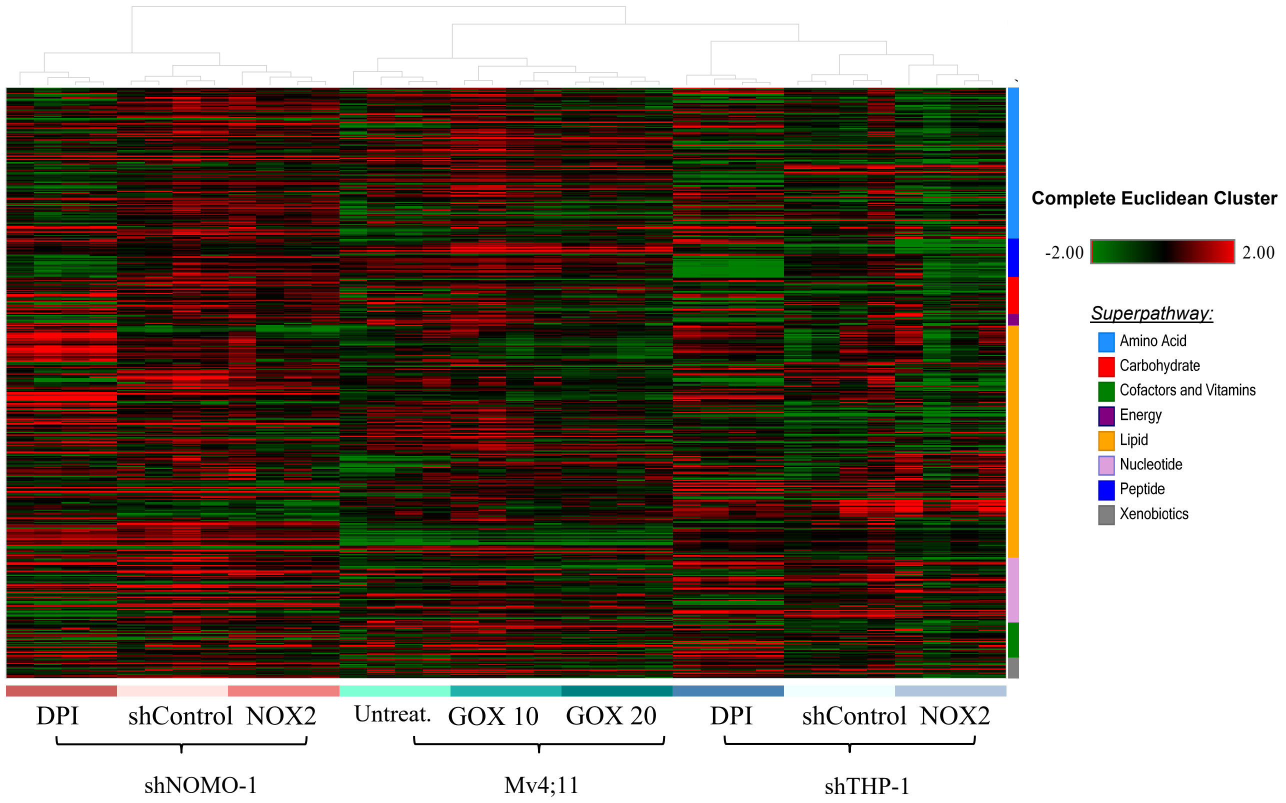

Supplemental Figure S2. Variation in biochemical metabolites between samples analyzed by Metabolon™. Dendrogram representing variation between samples subjected to hierarchical clustering analysis. Samples were loaded equally across the platform, normalized to Bradford protein concentration and each biochemical rescaled to set the median equal to l, imputation of missing values (if any) with minimum value for each compound is performed and data log transformed. Green represents lower and red higher concentration of biochemical relative to median value. Each experimental condition was replicated (n=4) and is color coded on the x axis.

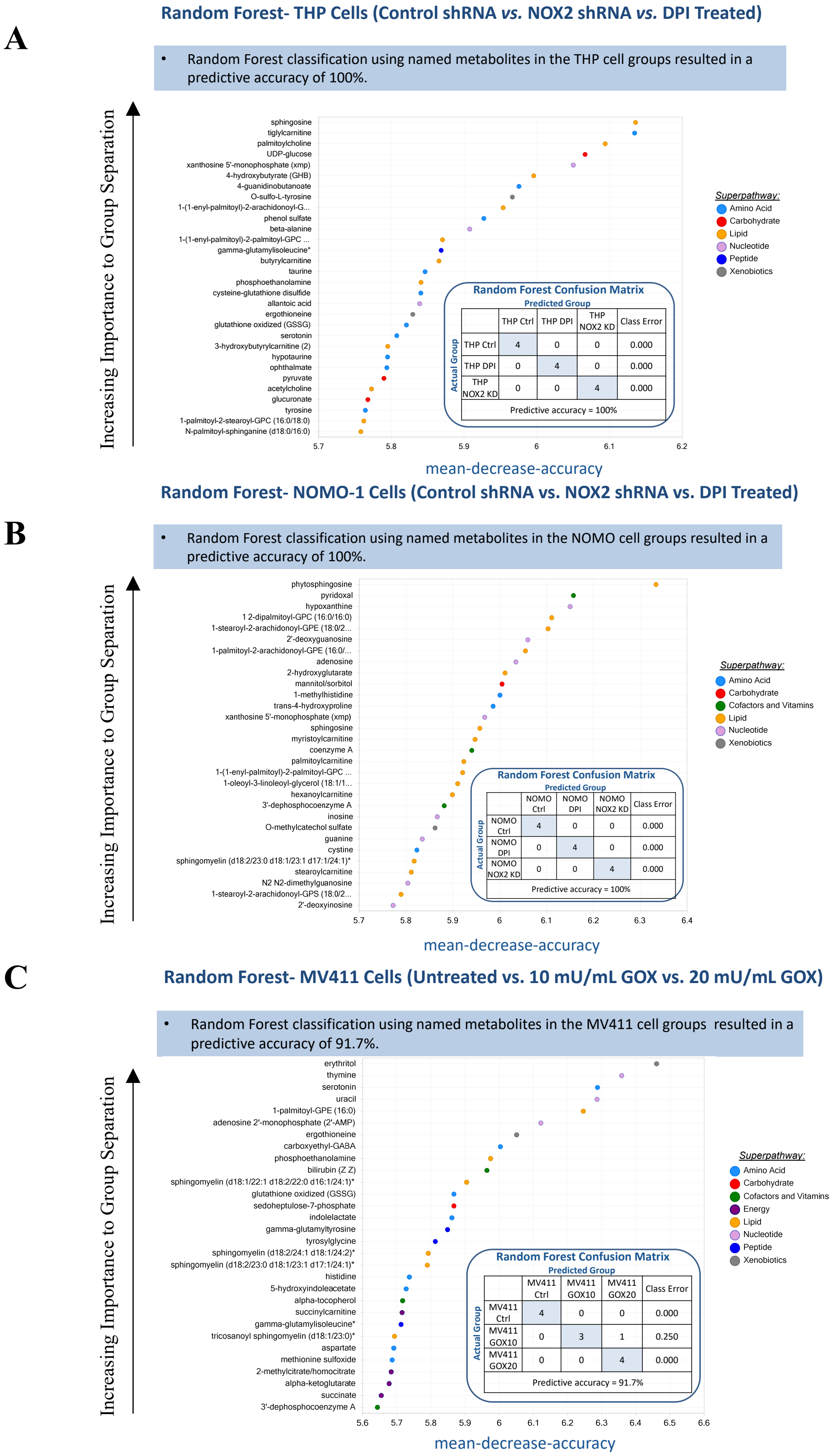

Supplemental Figure S3. Random Forest analyses of AML cell lines exposed to different ROS levels. Random Forest classification of **(A)** THP-1 and **(B)** NOMO-1; in which cells were treated with 100nM DPI for 24 h or NOX2 was knocked down. Control cells were shTHP-1 or shNOMO-1 **(C)** MV4;11 cells were cultures with increasing concentrations of H_2O_2 (GOX). The predictive accuracy is based on their metabolic profiles which is better than random chance alone (33% accuracy for three groups), indicating that differences in biochemical profiles between groups may be sufficient for biomarker discovery.

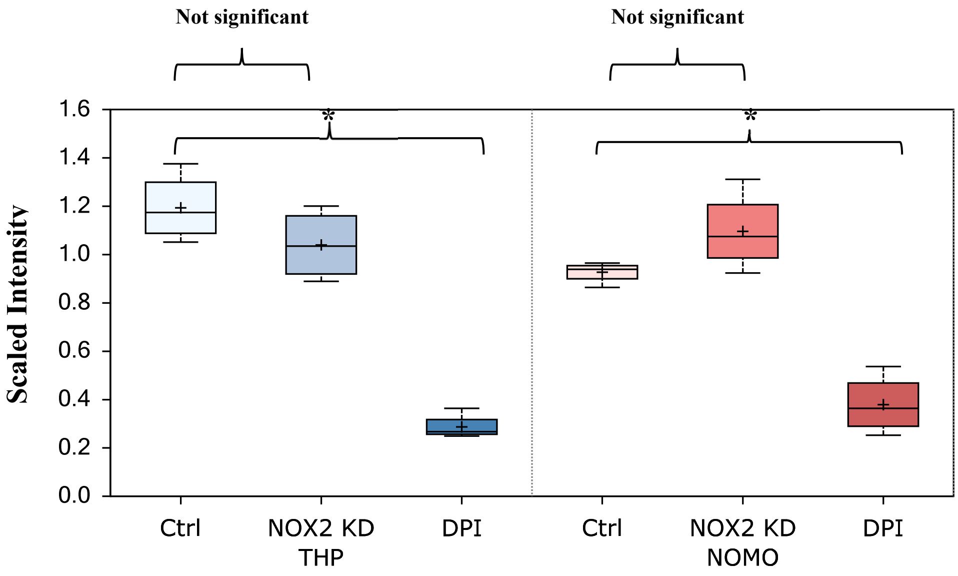

Supplemental Figure S4. Alterations in fumurate biochemicals in NOX2 KD and DPI-Treated AML cell lines. Data from global biochemical profiling of shTHP-1 and shNOMO-1 cells with NOX2 KD or treated with DPI (100 nM) for 24 h followed by analysis by Metabolon™. Levels of biochemicals normalized to total protein in purine metabolism. Statistical significance analyzed by Welch's two sample t-Test (n=4 per group) and significance denoted by *, $P<0.05$. Ctrl, vehicle treated control cells.
